# Diffuse Gonococcus Infection

**Published:** 1903-10-31

**Authors:** 


### DIFFUSE GONOCOCCUS INFECTION.

It is gradually becoming more generally appreciated that secondary manifestations of gonorrhœa may affect almost any tissue of the body, although the joints are the commonest parts to be affected in this way. An interesting case which shows an unusual form of metastatic inflammation due to gonococcus infection is reported by Dr. Charles A. Powers.[1] The patient was a man, 28 years of age. On the ninth day of an ordinary uncomplicated attack of gonorrhœal urethritis he complained of a diffuse pain in and about the right elbow. The elbow was found to be moderately swollen, and the swelling and pain rapidly increased and spread up and down the limb. The patient felt ill and remained in bed, the temperature being raised and the arm being extremely painful. Four days after the commencement of the swelling it had extended up to the shoulder and down to the wrist, and at the level of the elbow the circumference of the right arm was two and a half times that of the left. Fluctuation could not be felt. There was no history of an injury and a minute examination of the entire limb failed to reveal evidence of a scratch or other small breach of skin. It was impossible to say whether or not the joint was affected. Two days later the condition having become steadily worse, free incisions were made, the patient being under ether anæsthesia. These incisions liberated a considerable amount of thin clear fluid, pus being found at one spot just below and in front of the external condyle of the humerus. The elbow joint did not appear to be distended. Coverslip preparations of the clear fluid showed the gonococcus, pure cultures of which were grown from the same source by the method of Wasserman. The pus showed the presence of staphylococci as well as of gonococci. The latter were also demonstrated in the urethral pus. Two days after the operation the whole neck swelled and became very tender and painful, the pulse increased in frequency to 100, the heart sounds became very soft and indistinct, and it seemed as though a general gonococcus infection had ensued. The next day the left parotid gland was swollen and painful, and the right shoulder, and pectoral regions were involved. The condition of the patient seemed very grave. However, he began to improve, the swelling diminished, and convalescence was soon established. The wounds healed, but the elbow and wrist joints remained stiff in spite of every care, and two years after the illness the right elbow joint was almost fixed.

[1] Medical Record, October 3.

